# Systematical identifications of prognostic meaningful lung adenocarcinoma subtypes and the underlying mutational and expressional characters

**DOI:** 10.1186/s12885-019-6462-y

**Published:** 2020-01-27

**Authors:** Zhenyang Lv, Ting Lei

**Affiliations:** The Second Affiliated Hospital of Dalian Medical University, Dalian, Liaoning 116000 People’s Republic of China

**Keywords:** Lung adenocarcinoma, Prognosis, Causal regulatory network, Prognosis master regulator, Subtypes

## Abstract

**Background:**

Lung adenocarcinoma (LUAD) is one of the most common cancer types, threatening the human health around the world. However, the high heterogeneity and complexity of LUAD limit the benefits of targeted therapies. This study aimed to identify the key prognosis impacting genes and relevant subtypes for LUAD.

**Methods:**

We recognized significant mutations and prognosis-relevant genes based on the omics data of 515 LUAD samples from The Cancer Genome Atlas. Mutation significance was estimated by MutSigCV. Prognosis analysis was based on the cox proportional hazards regression (Coxph) model. Specifically, the Coxph model was combined with a causal regulatory network to help reveal which genes play master roles among numerous prognosis impacting genes. Based on expressional profiles of the master genes, LUAD patients were clustered into different sub-types by a consensus clustering method and the importance of master genes were further evaluated by random forest.

**Results:**

Significant mutations did not influence the prognosis directly. However, a collection of prognosis relevant genes were recognized, where 75 genes like *GAPDH* and *GGA2* which are involved in mTOR signaling, lysosome or other key pathways are further identified as the master ones. Interestingly, the master gene expressions help separate LUAD patients into two sub-types displaying remarkable differences in expressional profiles, prognostic outcomes and genomic mutations in certain genes, like *SMARCA4* and *COL11A1*. Meanwhile, the subtypes were re-discovered from two additional LUAD cohorts based on the top-10 important master genes.

**Conclusions:**

This study can promote precision treatment of LUAD by providing a comprehensive description on the key prognosis-relevant genes and an alternative way to classify LUAD subtypes.

## Background

Lung cancer is one of the most frequent malignant neoplasm, and one of the major causes of cancer death among both males and females around the world [1, 2]. Lung cancer is a highly heterogeneous and complex disease which includes many subtypes. Histologically, lung adenocarcinoma (LUAD) is the most common one. Recently, molecular targeted therapies have improved the treatments for LUAD, particularly for patients with specific mutations in *EGFR* [3], *ALK* [4, 5], *RET* [6] and *ROS1* [7]. Meanwhile, promising novel targets like *KRAS* [8] and *MET* [9] are being studied. However, the high heterogeneity and complicated molecular patterns of LUAD limit the benefits of these targeted therapies to only specific patients, leaving large amount of LUAD patients without effective therapeutic drugs. It is essential to obtain a more comprehensive view on the molecular mechanism of LUAD, rather than solely focusing on the therapeutically targeted mutations.

Owing to the advantage of high-throughput omics technology, large scale descriptions on the molecular characters of LUAD have been achieved [10]. Accordingly, the potential complicated molecular mechanism underlying LUAD has been more extensively explored by mining the LUAD relevant omics data [11] [12]. These omics based studies help identified a series of prognosis or diagnosis relevant biomarkers which can provide novel and promising treatment targets. However, the omics-based cancer investigations, which mainly depend on mutation significance examination, differential analysis, or expression-based survival analysis, will generate a larger number of interesting items, either in gene or protein level [10]. It is unquestionable these genome- or proteome-wise identifications generate certain mechanical or clinical meaningful biomarkers [13, 14]. However, human body is a complex organism, these interesting items must function in a collective way rather than individually. A big challenge is how to understand the mutual associations among these most functional items and recognize the most functional multi-item sets from the interesting items. Besides, the consistency of the identified molecular patterns across different datasets is also an important issue.

Here, we put-forward a causal network based framework to systematically investigate on the prognosis-relevant genes and their mutual association patterns underlying LUAD. Through this study, a causal regulatory network among prognosis relevant genes will be constructed. Based on this network, we can identify the master prognosis impacting genes and the prognosis-meaningful LUAD subtypes can be recognized.

## Methods

### The Cancer genome atlas (TCGA) data

The mutation and RNA-seq data for LUAD were obtained from TCGA [15]. Firstly, we downloaded both kinds of data for 33 types of cancers from the National Cancer Institute’s Genomic Data Commons (GDC) (https://gdc.cancer.gov/about-data/publications/pancanatlas). The mutational data were saved in mutation annotation format [16], and the RNA-seq data were saved in a tab file. The maf data was processed by the R package maftools, and the RNA-seq data were preprocessed based on the voom algorithm [17] in the R package limma [18]. For this study, we extracted the data corresponding to LUAD patients.

### Pathway data

Pathway information were integrated from two databases including Kyoto Encyclopedia of Genes and Genomes (KEGG) and Molecular Signatures Database (MsigDB, http://software.broadinstitute.org/gsea/msigdb) [19], and pathway names as well as genes belonging to each pathway were extracted from the databases.

### Identification of significant somatic mutated genes (SMGs)

MutSigCV(version 1.3.4) [20] was applied on the the maf mutation file to recognize significant SMGs where the significance threshold was set as q-value < 0.1. Then, we utilized the maftools to visualize the mutation information of these significant SMGs among TCGA LUAD patients.

### Survival analysis based on gene expressions

The clinical information of all TCGA-LUAD patients was also obtained from the GDC. For the mRNA expression data, we removed genes with more than 70% of zero values, and analyzed the prognosis impacts for the remained genes. For each such gene, we utilized a Cox proportional hazards (Coxph) regression model in the R package “survival” [21] to examine whether the expression level of this gene has a significant influence on the survival rate. According to the Coxph results, genes with *p*-values less than 0.05 were regarded as prognosis-relevant, and if the regression coefficients are larger than 0, then higher expression levels will correspond to worse survival rates, otherwise, higher expression levels will correspond to better survival rates.

### Identification of master prognosis impacting genes by a causal regulatory network

According to the Coxph-based survival analysis results, genes with *p*-values less than 0.01 and absolute coefficient values larger than 0.2 were taken as the prognosis relevant genes. Then, based on the mRNA expression profiles of these genes, we computed the bi-weight mid-correlations [22] among all pair-wise genes. To recognize the most likely causal correlations, we further estimated a causal regulatory network based on the correlation matrix. This causal regulatory network was a directed acyclic graph describing the conditional independence relationships. It was estimated by the PC-algorithm (named after its inventors Peter Spirtes and Clark Glymour) in R package pcalg [23]. Since this causal regulatory network was directed, a summarized node degree was calculated as the number of all out-going edges minus the number of all in-coming edges (i.e., out degree - in degree). Then nodes were taken as the master prognosis impacting genes if the absolute values of their summarized degrees were larger than certain threshold.

### Unsupervised clustering of patients based on SMG relevant genes and pathways

We clustered the LUAD patients into two groups based on the mRNA-level expression matrix of the master prognosis regulating genes. This expression matrix was scaled by subtracting the mean level and being divided by the standard derivation with respect to each individual gene. Based on the scaled expression matrix, we applied a consensus clustering method in the R package “ConsensusClusterPlus” [24] to cluster the patients into 2 clusters where “partitioning around medoids” was chosen as the basic clustering algorithm.

To further examine the significance of the prognosis effects generated by the two clusters, we also repeated the clustering processes 100 times, where each time we clustered the LUAD based on the expression profiles of randomly-selected genes (with the same number of the prognosis-relevant genes). Thus, we obtained the distribution of the log-rank *p* values for randomly conditions.

### Evaluate the importance of genes for the clustering results

After clustering the patients into 2 clusters, we used the random forest (RF) [25] algorithm to evaluate the importance of all genes in the input expression matrix for predicting the accurate cluster labels. These genes were ranked by the importance score. Besides, we also examined enrichment significance of the top-50 important genes in each pathway based on the hypergeometric distribution.

### Estimate the relevance between the prognosis relevant genes and SMGs

For each SMG, we separated the samples into mutated and wild type sets, and utilized T-test (un-paired, two-sides) to identify which genes were differentially expressed between mutated and wild type set in the transcriptomics data, then genes with FDR less than 0.1 were regarded as the SMG relevant.

### Predicting and validating the LUAD subtypes based on the other independent lung cancer cohorts

The mRNA expression matrix and the corresponding clinical information for two lung cancer cohorts (GSE30219 and GSE31210) were downloaded from Gene Expression Omnibus (GEO) database by the R package ‘GEOquery’ [26]. We constructed a cluster-label predictor based on the TCGA-LUAD expression matrix of the top-10 important genes. This predictor was trained by a lasso and elastic-net regularized generalized linear model (termed as glmnet, implemented by the R package “glmnet” [27]). Firstly, we estimated the leave-one-out validation accuracy of the top-10 important genes in predicting the subtypes based on the LUAD transcriptomics data. During leave-one-out validation, each individual sample was respectively taken out as a testing sample, and the other samples were taken as the training samples, then a glmnet-based subtype-predictor was trained and tested by the training and testing samples, when all samples were utilized as a testing sample for once, the ratio of corrected predicted times was regarded as the model accuracy. Then, we used the TCGA LUAD data to train a final predictor and applied this predictor on the GEO datasets to predict the corresponding cluster-labels for each patient, and survival differences of the two predicted clusters were tested by Coxph model and the corresponding survival curves were estimated by the Kaplan-Meier method.

### Drug and gene interactions

All drug and gene interaction information were obtained from the DGIdb [28] which included both the known and reported drug-gene interactions.

### Statistical analysis

The statistical analysis and relevant computations were implemented by R. Detailed information was described in corresponding method sections.

## Results

### Significant somatic mutations in LUAD

**Fig. 1:**
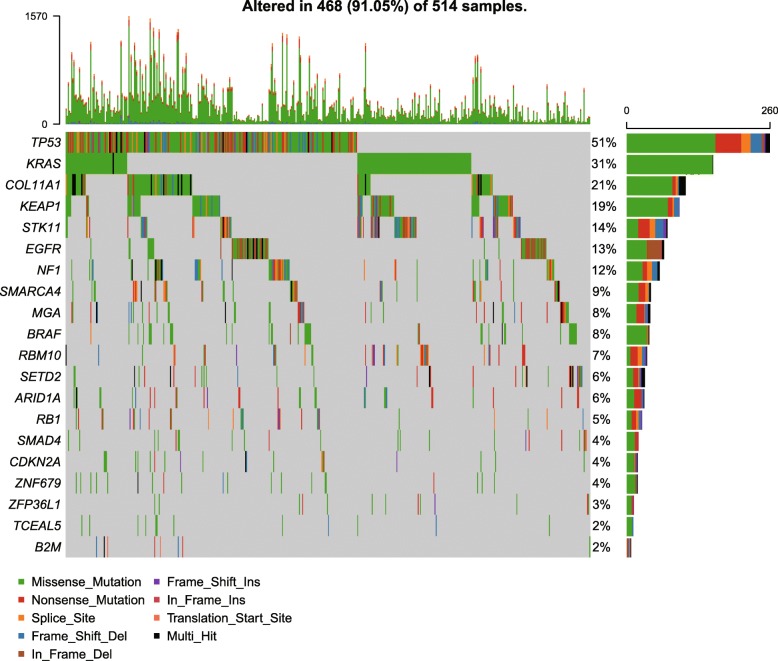
Significant somatic gene mutations in lung cancer. Different colors stand for different mutation types

According to the gene mutation data of 515 LUAD patients in TCGA [29], we identified 20 significant SMGs by MutSigCV (q-value < 0.1, Fig. 1). Most of these SMGs like *TP53*, *KRAS*, *KEAP1*, *STK11*, *EGFR*, *NF1*, *BRAF*, *SMARCA4*, etc. have already been identified in the other studies, especially the study conducted on the previously collected 230 TCGA-LUAD patients [10]. The significant mutations of *COL11A1* (q-value = 8.8e-06, mutation rate = 21%) is rarely identified in the previous LUAD studies. However, expressions of *COL11A1* have shown associations with prognostic factors, pathological stages, and lymph node metastasis in non-small cell lung cancer (NSCLC) in some previous studies [30–33]. The mutations of *COL11A1* might also be one of the driven factors for LUAD.

To evaluate the most direct mutational effects, we compared the expressions of these SMGs in the mutated and wild type samples. We found that some of the SMGs can lead to significant expressional alterations (Table 1). For instance, the mutations of *RBM10* will significantly reduce its expressions while mutations of *EGFR* might improve the expression levels (see Additional file 1). However, only three of the expressional alterations (*SMAD4*, *CDKN2A* and *TCEAL5*) can lead to significant prognosis influence (see Additional file 1). Besides, we also examined whether the mutations can affect the expressions of the other genes. We found that most of the mutations were related with significant expressional alterations of various genes (see Additional file 2), e.g., KEAP1 mutations are related with the up-regulated mRNA expressions of G6PPD, TRIM16, GCLM, etc., implying that these mutations may lead to remarkable down-stream alterations in the mRNA level.

**Table 1:** The expressional alterations of mutated genes

Gene	Fold Change	*P*-value
KRAS	1.09	1.5E-16
SMAD4	0.92	8.4E-4
SMARCA4	0.86	1.2E-4
STK11	0.92	3.1E-7
EGFR	1.24	3.0E-13
KEAP1	1.04	4.2E-7
TP53	0.96	2.1E-3
RB1	0.93	2.9E-4
RBM10	0.79	5.5E-12
COL11A1	1.26	1.4E-2
NF1	0.94	3.4E-3
SETD2	0.97	1.2E-2
CDKN2A	1.93	9.8E-7
ARID1A	1.00	0.85
B2M	0.96	0.26
BRAF	1.02	0.32
TCEAL5	1.06	0.97
ZFP36L1	1.04	0.15
MGA	0.95	0.097
ZNF679	0.93	0.16

Note: Fold change: examine the fold change in mRNA expression level of one gene between samples with mutated and wild type gene status; *P*-value: Wilcox-test, unpaired, two sided

### Genome-wide identification of prognosis relevant genes for LUAD

**Fig. 2:**
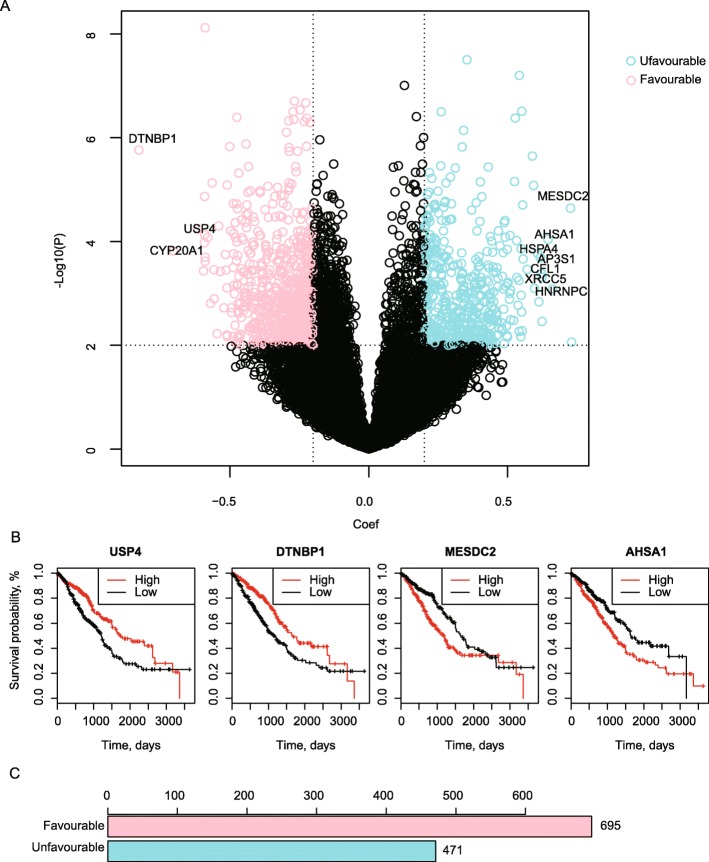
Genome wide prognostic analysis results. **a** A volcano plot showing the genome wide prognosis analysis results. The vertical and horizontal axis respectively represent the –log10 (p) and regression coefficient value (coef) got from the Coxph survival analysis. Each data point represents the Coxph analysis result of one gene in the mRNA level. Coef < 0 means hazard ratio (HR) < 1 and coef > 0 means HR > 1. Strictly, only genes with *p* < 0.01 and coef > 0.2 were regarded as unfavourable for prognosis while genes with *p* < 0.01 and coef <− 0.2 were regared as favourable. **b** The number of significant favourable and unfavourable genes. **c** Survival curves for examples of favourable and unfavourable genes

The genomic mutations can not only influence the functions of the mutated genes, but may also generate remarkable effects on down-stream cascades, thus leading to the final impacts on clinical phenotypes, like the prognostic outcomes. To gain a comprehensive understanding on the prognosis impacts, we attempted to get a genome-wide estimation of the prognosis relevant genes for LUAD based on a Coxph model (see Methods). Through this analysis, we observed that a great number of genes were associated with LUAD prognosis (Fig. 2a). The high expression levels of some genes like *USP4*, *DTNBP1* may contribute to better survival rates (these genes were termed as favourable ones), while some of the others (termed as un-favourable ones) such as *AHSA1* and *MESDC2* may lead to worse survivals (Fig. 2a and b). AHSA1 is a co-chaperone of HSP90AA1, and a previous study has revealed that it is involved in the proliferation, migration, and invasion processes of osteosarcoma [34]. Here, we observed that higher expression of AHSA1 was associated with a worse survival rate (Fig. 2b). USP4 is a deubiquitinating enzyme which may inhibit p53 by deubiquitinating an important p53 ubiquitin ligase ARF-BP1 [35]. Correspondingly, many studies have identified the oncogene effects of USP4 [36]. Here, on the contrary, we found that higher expressions of USP4 in LUAD may lead to better prognosis. This may be caused by the highly heterogeneity of cancers and the alternative deubiquitinating substrates for USP4. Consequently, the prognosis effects of these identified genes may be conditional.

Meanwhile, although more favourable genes were observed than un-favourable ones, both kinds of genes exhibited a large exploring space (Fig. 2c). This indicates the complicated molecular patterns underlying LUAD pathology and implies the importance to identify the most meaningful genes which may play the master roles in regulating the disease progression.

### Construction of a potential causal regulatory network for prognosis-relevant genes

**Fig. 3:**
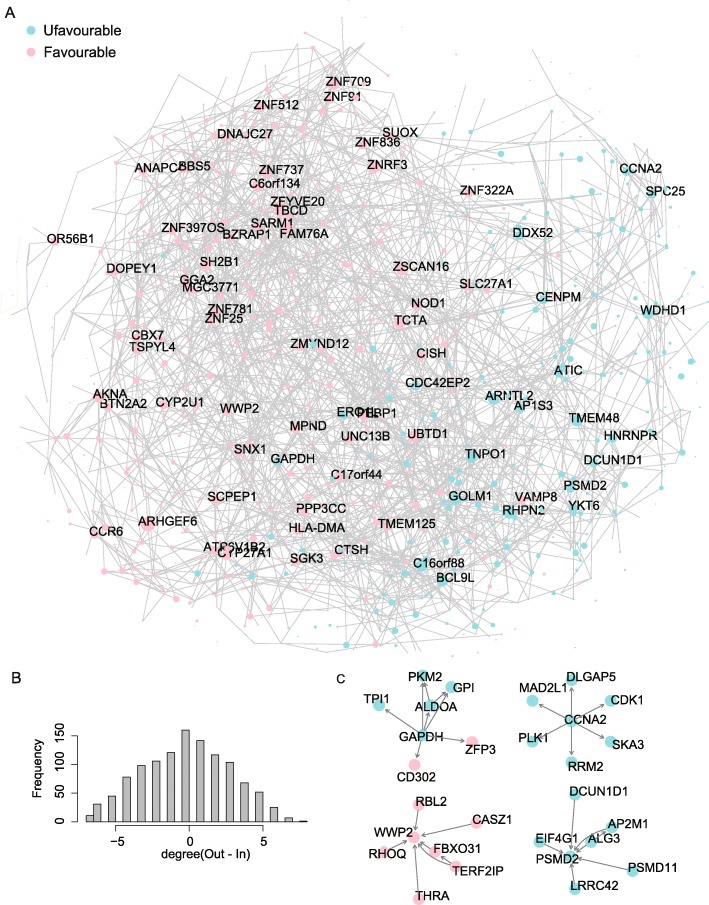
A causal regulatory network for prognosis relevant network. **a** The prognosis relevant causal regulatory network, where nodes represent genes and edges represent potential causal correlations. For clarity, only nodes with degrees larger than 5 are displayed. **b** Degree distribution of the nodes. **c** Representative sub-graphs

To understand the relations among all of the prognosis relevant genes and to help identify the master prognosis-influencing genes, a potential causal regulatory network was estimated (see Methods). Nodes in the network were all with significant prognostic impacts (*P* < 0.01 and | coef | > 0.2 based on Cox survival analysis) (Fig. 3a). The causal structure of this network was inferred from the transcriptional expressions of genes across TCGA-LUAD patients, where directed edges describe the identified direct causal effects, and bi-directed edges represent uncertainty in the constructed network [23]. According to this network, we found that most of the nodes with the same type of prognosis impacts (either favourable or un-favourable) gathered together while nodes with reversed impacts were relatively separated in the network. Since the network described the potential causal structure, the survival impacts of many nodes may be in-directly generated from their up-stream or down-stream items. Consequently, it is more important to identify which prognosis-relevant genes play the master roles rather than just examining the prognosis relevance.

To distinguish the master regulators, we further investigated on the node importance in the causal network. Here we calculated the summarized node degree by subtracting the number of in-coming edges from the number of out-coming edges for certain node. We found that the summarized degrees for a large portion of nodes were within the range of − 5 to 5 (Fig. 3b). The other nodes which were with larger absolute values of summarized degrees were hub nodes of the network (only names of the hub nodes were displayed on Fig. 3a). These nodes are more likely to play master roles in influencing LUAD prognosis, since the expression alterations of multiple prognosis relevant genes were highly associated with these hub nodes. For instance, GAPDH, CCNA2, WWP2 and PSMD2 were all hub nodes (summarized degree > 5 or summarized degree <− 5), and they may lead to remarkable prognosis influences by either passing its alteration to a series of down-stream partners (see sub-graphs for GAPDH and CCNA2, Fig. 3c) or gathering the influences from a collection of up-stream elements (see sub-graphs for WWP2 and PSMD2, Fig. 3c). The network structure may also indicate potential regulatory relationships between nodes. GAPDH, ALDOA and PKM2 all play functions in glycolysis [37], here, we also observed that they aggregated in the same sub-graph, implying that the data-driven network may suggest meaningful biological associations.

### Clustering analysis reveals LUAD-subtypes

**Fig. 4:**
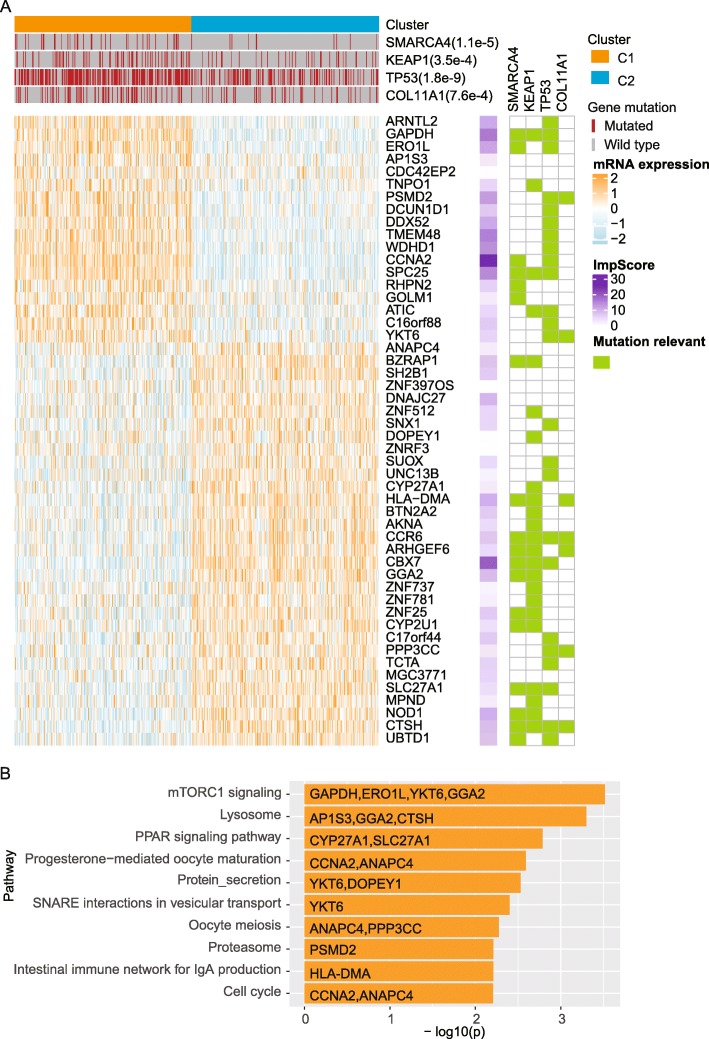
Clustering patients into subtypes based on high degree nodes in the causal regulator network. (A) A heatmap showing the expression profiles of the top-50 important genes for the clustering results. Above the heatmap, annotations about the clustering labels and SMGs which showed significant enrichment in one of the cluster were shown. On the right side of the heatmap, the first column represents the importance score of each gene, and the other columns stand for the relevancies between the genes and SMGs where a green grid means that expressions of the gene is with significant difference between the SMG mutated and wild type samples. (B) Pathway enrichment analysis results for the top-50 important genes

To understand the whole molecular and prognostic impact generated by the above identified master prognosis impacting genes, we clustered the LUAD patients into two groups (termed as C1 and C2 respectively) based on the mRNA-level expressions of all of the hub nodes in the causal regulatory network. We further evaluated the importance of each master gene, the top-10 important master genes for the two subtypes included *CCNA2*, *CBX7*, *TMEM48*, *SPC25*, *GAPDH*, *WDHD1*, *PSMD2*, *ERO1L*, *DDX52*, *ARNTL2* (Fig. 4a, only the top-50 important genes were shown in the central heat map). These two clusters showed significantly different expressional patterns, especially for genes in the mTOR signaling pathway, lysosome, and PPRA signaling pathways (Fig. 4b).

**Fig. 5:**
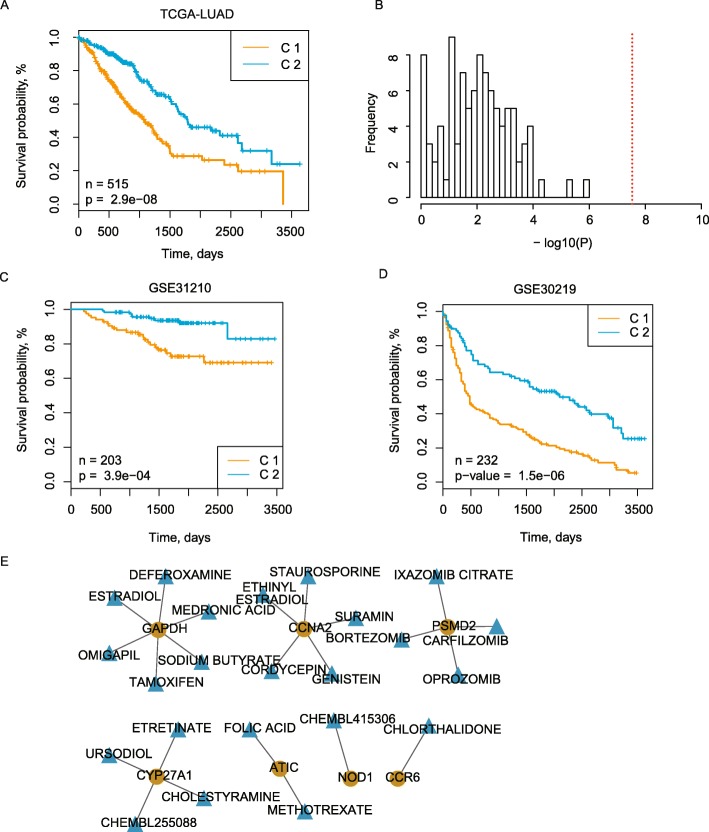
alidate prognosis relevant expression profiles in independent LUAD cohorts. **a** A Kaplan-Meier (KM) plot of the survival curves for the two estimated clusters. **b** Distribution of the –log10 transformed log-rank *p*-values for comparing the survival rates between two clusters identified based on randomly-selected genes. The red line represents the –log10 (p) got in (**a**). (**c**-**d**) Kaplan-Meier (KM) plots of the survival curves for two predicted patient clusters in GSE31210 (**c**) and GSE30219 (**d**). (**e**). Drug interaction network. Blue and yellow nodes respectively represent drugs and genes. Edges mean the gene/protein is one of the reported targets for the connected drug

Meanwhile, potential SMGs which may be related with the expressional differences between the two identified clusters were also identified. Mutations of *SMARCA4*, *KEAP1*, *TP53* or *COL11A1* were significantly enriched in C1 (Fig. 4a). These mutations were related with the significantly differential expressions for specific genes across the two clusters. For instance, *GAPDH* was highly expressed in C1, and its expression levels in patients with mutations in *TP53*, *KEAP1* or *SMARCA4* were significantly higher than wild type patients (Fig. 4a). Notably, we also observed that patients in C1 were with significantly worse prognosis than C2 (Fig. 5a, *P*-value = 2.9*10^− 8^), and the significance was more remarkable than those obtained from random gene sets with the same number of genes (Fig. 5b). Taken together, the master prognosis regulating genes help identify two meaningful subtypes for LUAD which showed significantly differential patterns in genomic and transcriptional levels.

### Validating the prognosis differences between the identified LUAD subtypes

To estimate the reliability and robustness of the identified LUAD subtypes, we also utilized additional two GEO datasets to further validate the expressional and prognostic patterns of these two subtypes. Firstly, we estimated the accuracy of top-10 genes in predicting the subtypes by leave-one-out cross-over validation based on the glmnet algorithm. The leave-one-out validation showed these top-10 items can help predict the subtypes with a 90% accuracy (see Additional file 3). Accordingly, a subtype predictor was trained based on the expressional profiles of the identified top-10 important master genes in the TCGA-LUAD cohorts (see details in Methods). Based on this predictor, other patients having similar expressional profiles with C1 or C2 will be annotated with the corresponding sub-type labels. Then, this predictor was applied on two independent LUAD cohorts (GSE30219 and GSE31210). Thus, patients in the independent cohort were also annotated into the two subtypes (C1 and C2) based on their expressional profiles, and survival analysis showed these two groups were also with significantly differential survival outcomes just as observed in TCGA-LUAD, where C1 was with significantly worse prognosis than C2 (Fig. 5c and d). This verifies the robustness of the identified expression-based LUAD subtypes in independent cohorts.

### Promising drugs for the identified LUAD-subtypes

Since the survival differences were highly related with the expressional alterations in the identified clusters, drugs targeting on the most important genes for classifying the two subtypes may generate distinctive effects on the two subtypes. Based on this hypothesis, we attempted to obtain potential drug-gene interactions for the top-30 important genes (see Methods). As results, we found a collection of drugs like Estriol, Ethinyl and Folic acid [28] may be associated with genes like *GAPDH*, *CCNA2* and *PSMD2* (Fig. 5e, and three of them were among the top-10 important genes for the two subtypes) which may be highly contributable to the survival differences between the two identified subtypes. For LUAD patients, more attention should be paid on these drugs since patients from different subtypes may have distinctive responses to these drugs.

## Discussion

LUAD is one of the most common cancer types, threatening the human health around the world. The development of targeted therapies, especially those targeting on *EGFR* [3] and *ALK* [5], have promoted the treatment of some LUAD patients, however, the highly heterogeneity of LUAD makes the benefits of these therapies limited to few patients. Through this study, prognostic meaningful lung adenocarcinoma subtypes which are independent of *EGFR* and *ALK* mutations and the relevant mutational and expressional profiles were identified. We provide an alternative way to classify the LUAD subtypes which showed differential transcription profiles and remarkable prognosis differences, and provide promising prognostic biomarkers for the subtypes.

With the development of high-throughput biological and chemical technology, a great deal of omics-data is accumulated to help describe the molecular mechanisms of different types of cancers. Owing to omics-data measured for LUAD cohorts [15, 34, 38], a large number of significantly mutated, prognosis-relevant or differentially expressed genes (e.g., *EGFR*, *TP53*, *KRAS*) for LUAD can be identified. However, the high heterogeneity and complicated molecular patterns of LUAD makes it insufficient only fucosing on these limited hallmark genes. It is essential to obtain a more comprehensive view on the molecular mechanism of LUAD, rather than solely focusing on the hallmark mutations. Omics data provide a valuable resource to identify potential prognosis relevent genes. However, tranditional survival analysis always led to a large number of statistically significant genes where many indirect ones were mixed in. In this study, we not only identified the prognosis-relevant genes, most of which are independent of the LUAD hallmark mutations (e.g., *EGFR*, *KRAS*, *ALK*), but also constructed the potential causal regulating structures among these genes, thus identifying which genes are more likely to play master roles in influencing the LUAD patients’ prognosis in the transcriptional level, providing more new promising therapeutic targets for the heterogeneous LUAD patitens. Based on these master genes, we also identified two potential LUAD subtypes. The poor survival rate of one sub-type may be related with mutations in *SMARCA4*, *KEAP1*, *TP53* and *COL11A1*. Low expression of SMARCA4 has been reported to be significantly associated with poor prognosis and can be served as a predictive biomarker of increased sensitivity to platinum-based therapies [39]. Here, the significant mutations of SMARCA4 were also related with the poor survival rate of one LUAD subtype (Fig. 4), and the mutations may lead to decreased expressions of SMARCA4 (see Additional file 1). Similarly, KEAP1 [40], TP53 [41] and COL11A1 [33] have all been reported to play roles in LUAD. Co-occurrence of these SMGs in the poor survival subtype implies that the differential prognosis between the two subtypes is not simply the result of one specific gene but a collection of meaningful genes. Simply targetting on one specific SMG is not sufficient to resist the disease progression, alternative treatment targets, like key downstream elements, should also be considered. Accordingly, a collection of drugs like Estriol, Ethinyl and Folic acid [28] are identified as promising drugs for the identifed poor prognosis subtypes, these drugs can target on genes which may be highly contributable to the survival differences between the two identified subtypes like *GAPDH*, *CCNA2* and *PSMD2*. Meanwhile, the molecular mechanism underlying the two sub-types is associated with multiple down-stream pathways, e.g., mTOR signaling pathway and lysosome.

An important issue of omics-based cancer studies is whether the revealed results can be re-discovered in the other independent cohorts despite cancer heterogeneity or sample biases. Here, based on the expressional profile of master genes, the two identified subtypes were consistent in multiple independent cohorts, confirming the robustness of the identified subtypes which showed significant differences both molecularly and clinically. The robustness of the subtypes also imply that the causal regulatory network based method help identify the most influential genes. These results can provide an alternative way to classify LUAD patients and supply valuable references on selecting the most beneficial treatments for specific type of LUAD.

A limitation of this study is that most of the calculated relationships were significant in the statistical level. It is unavoidable that false positives are mixed into these statistical relations, e.g., the causal regulating effects. However, these findings still provide remarkable data resources, which may promote the discovery of promising molecular mechanisms underlying LUAD in a less time-and resource-consuming way. In the future research, more efforts will be put into validating these potential relations.

## Conclusions

This study provides a comprehensive description on the key prognosis-relevant genes for LUAD. We not only identify which genes are related with the LUAD prognostic outcomes, but also construct a potential causal gene regulatory network which may promote the understanding of meaningful biological associations among the numerous prognosis-relevant genes. We also put forward an alternative way to classify LUAD subtypes. This redefined LUAD subtyping strategy, validated by various independent cohorts, can help promote LUAD precision treatment. Taken together, this study describes the complicated molecular patterns underlying LUAD pathology to some degrees and provides guidance on the potential prognosis and subtyping biomarkers as well as future therapeutic targets for LUAD.

## Supplementary information

**Additional file 1:** Expressional alterations and clinical impacts of the significantly mutated genes. A. Boxplots of the expressions of SMGs in mutated and wild type tissues. B. Km-plots of SMGs with significant impacts on LUSC.

**Additional file 2:** Top-ranked differentially expressed genes between samples with and without certain mutations. For each SMG, we separated the samples into mutated and wild type sets, and examined which genes showed significant differential expressions in mRNA level between the mutated and wild type samples. SMGs (yellow nodes) and their relevant differentially expressed genes (blue nodes) are linked by edges. Red and green edge colors respectively represent positive and negative correlations, and the edge width is proportional to the absolute value of log2(Fold Change).

**Additional file 3:** The leave-one-out validation accuracy of the top-10 important genes for predicting the identified LUAD subtypes based on the glmnet algorithm. The best performance was achieved when lambda was set at 0. 0.007137788 and alpha was 1.00.

## Data Availability

The TCGA-LUAD data are available in the Genomic Data Commons (https://gdc.cancer.gov/about-data/publications/pancanatlas). The validation LUAD cohorts (GSE30219 and GSE31210) are available in GEO (https://www.ncbi.nlm.nih.gov/geo/query/acc.cgi?acc=GSE30219, https://www.ncbi.nlm.nih.gov/geo/query/acc.cgi?acc=GSE31210).

## Abbreviations

Coxph: Cox proportional hazards
GDC: Genomic data commons
GEO: Gene expression omnibus
KEGG: Kyoto encyclopedia of genes and genomes
LUAD: Lung adenocarcinoma
MsigDB: Molecular signatures database
RF: Random forest
SMG: Somatic mutated gene
TCGA: The cancer genome atlas
