# Supplementary figures and images for: Systematical identifications of prognostic meaningful lung adenocarcinoma subtypes and the underlying mutational and expressional characters
Source: BMC Cancer. 2020 Jan 27;20:56. doi: 10.1186/s12885-019-6462-y (PMC6983970; doi:10.1186/s12885-019-6462-y)

A

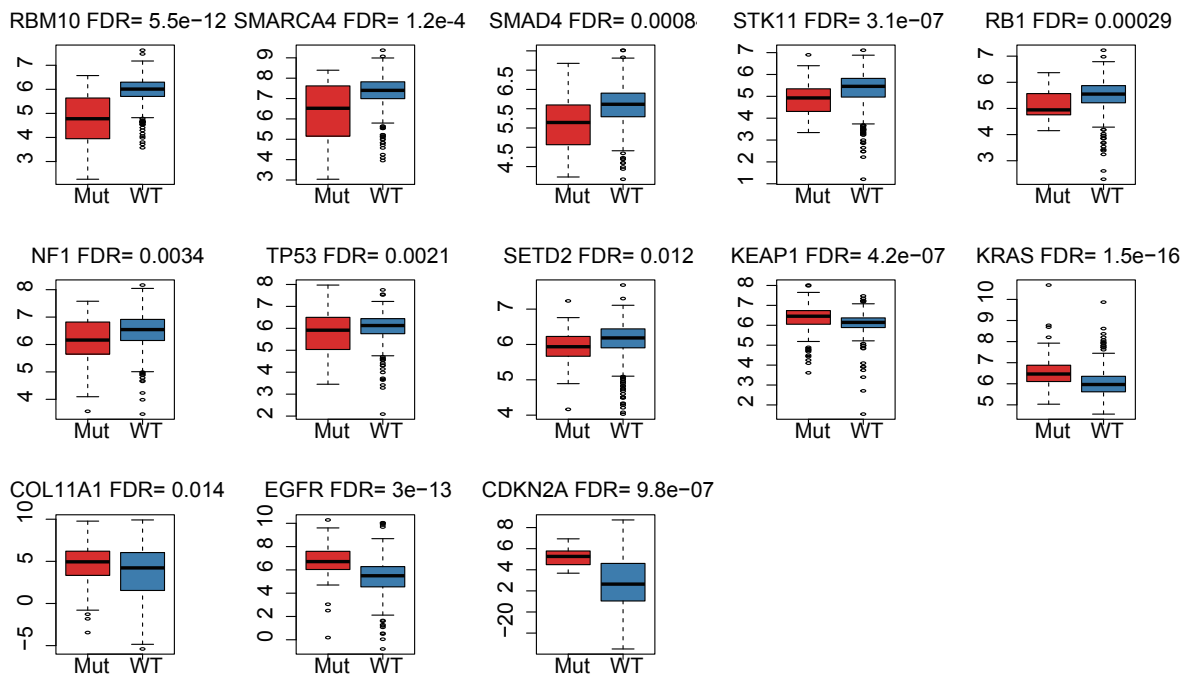

B

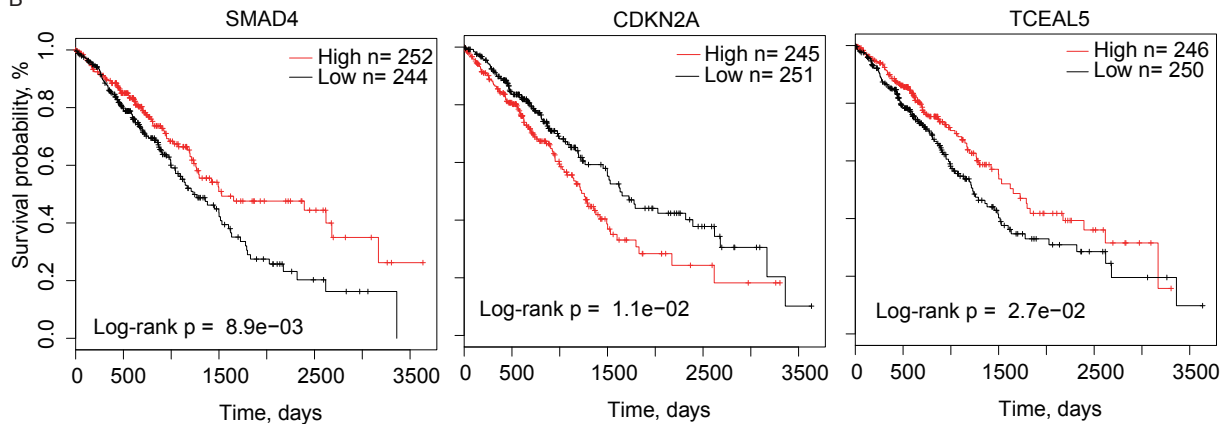

Supplement: Supplementary file 1 — Additional file 1: Expressional alterations and clinical impacts of the significantly mutated genes. A. Boxplots of the expressions of SMGs in mutated and wild type tissues. B. Km-plots of SMGs with significant impacts on LUSC. [file 12885_2019_6462_MOESM1_ESM.pdf]

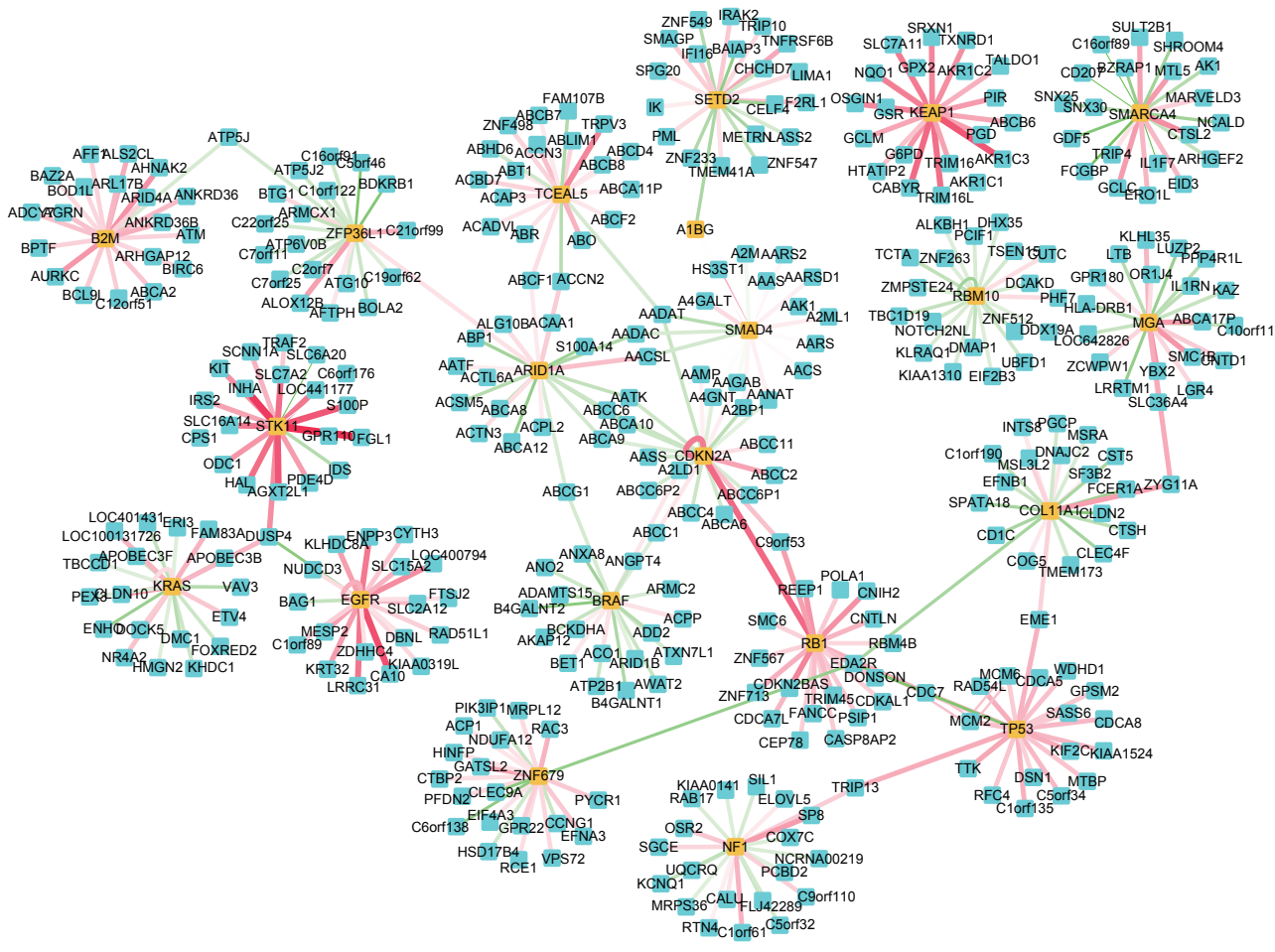

Supplement: Supplementary file 2 — Additional file 2: Top-ranked differentially expressed genes between samples with and without certain mutations. For each SMG, we separated the samples into mutated and wild type sets, and examined which genes showed significant differential expressions in mRNA level between the mutated and wild type samples. SMGs (yellow nodes) and their relevant differentially expressed genes (blue nodes) are linked by edges. Red and green edge colors respectively represent positive and negative correlations, and the edge width is proportional to the absolute value of log2(Fold Change). [file 12885_2019_6462_MOESM2_ESM.pdf]

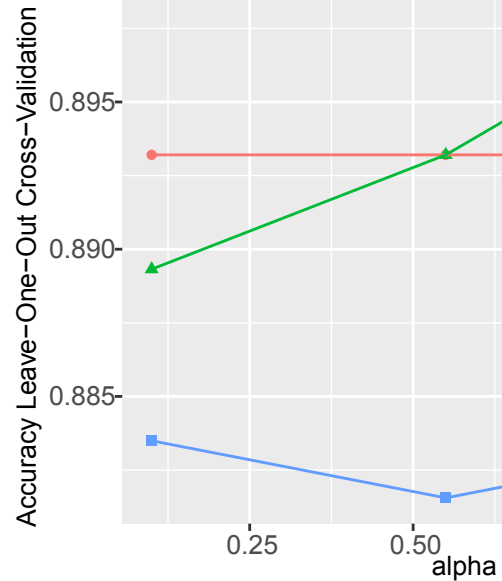

lambda

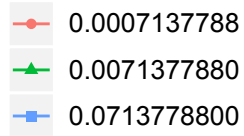

Supplement: Supplementary file 3 — Additional file 3: The leave-one-out validation accuracy of the top-10 important genes for predicting the identified LUAD subtypes based on the glmnet algorithm. The best performance was achieved when lambda was set at 0. 0.007137788 and alpha was 1.00. [file 12885_2019_6462_MOESM3_ESM.pdf]
